# The Frequency of Exfoliation Syndrome in the Central Anatolia Region of Turkey

**DOI:** 10.1155/2014/139826

**Published:** 2014-06-17

**Authors:** Raşit Kılıç, Hafize Sezer, Sebile Ü. Çomçalı, Serdar Bayraktar, Gökay Göktolga, Yasin Çakmak, Abdi B. Çetin, Tongabay Cumurcu

**Affiliations:** ^1^Department of Ophthalmology, Sivas Numune Hospital, Sivas, Turkey; ^2^Department of Biostatistics, Cumhuriyet University Faculty of Medicine, Sivas, Turkey; ^3^Department of Ophthalmology, Inönü University Faculty of Medicine, Malatya, Turkey

## Abstract

*Aim*. The aim of this study was to investigate the frequency of exfoliation syndrome in the Central Anatolia region of Turkey and to evaluate its relationship with cardiovascular and ocular diseases. *Methods*. Patients over the age of 45 years who presented to the clinic were included in the study. All cases underwent a comprehensive ophthalmology examination. Exfoliation syndrome was diagnosed with the presence of exfoliative material on the lens anterior capsule or iris on slit lamp examination. The patients were divided into two groups as the exfoliation syndrome group and nonexfoliation syndrome group according to the presence of exfoliative material. *Results*. Exfoliative material was found in one or both eyes of 212 of the 2103 patients (10.1%) evaluated within the scope of the study. A significant relationship was found between exfoliation syndrome and increasing age and male gender. A significant relationship was found between exfoliation syndrome and glaucoma, cataracts, age-related macular degeneration, and phacodonesis. While no relationship was found between exfoliation syndrome and hypertension or diabetes mellitus, a significant relationship was found with coronary artery disease. *Conclusion*. The unilateral or bilateral exfoliation syndrome frequency was 10.1% in this hospital-based study. A statistically significant relationship was found between exfoliation syndrome and advancing age, gender, and coronary artery disease.

## 1. Introduction

Exfoliation syndrome (XFS) is an age-related generalized disease of the extracellular matrix, characterised by the production and progressive accumulation of fibrillar extracellular material in many ocular and extraocular tissues, and is often associated with glaucoma [1]. Its frequency varies according to the ethnic population and geographic region. XFS causing an increase in trabecular outflow resistance is the most common cause of open-angle glaucoma [2]. Extracellular fibrillar material is widely present in the skin, lungs, liver, heart, gallbladder, blood vessels, and cerebral meninges [3]. XFS has been reported to be associated with systemic vascular diseases such as hypertension, angina, stroke, myocardial infarction, and abdominal aortic aneurysm [4, 5]. XFS also has nonophthalmic manifestations such as hyperhomocysteinemia and sensorineural hearing loss [6–9].

While there are two different epidemiological studies available from the Eastern Mediterranean (Adana Province) and Middle Black Sea (Tokat Province) regions of Turkey, no study has been conducted in Central Anatolia [10, 11]. In addition, the relationship of XFS with systemic diseases such as diabetes mellitus (DM), hypertension (HT), coronary artery disease (CAD), smoking, and alcohol intake has not been investigated in these previous two studies from Turkey. The relationship between cardiovascular diseases and XFS in Turkey has been investigated, but there are no relevant epidemiological studies [12, 13].

This study was carried out in Sivas, a province in the Central Anatolia region of Turkey, with a population of 623,000. Sivas has the highest number of villages in the country and is the second largest city of Turkey in terms of surface area. Besides, it is the second highest city of Central Anatolia (altitude: 1285 m). The Central Anatolia region receives one of the lowest amounts of rainfall in the country and has a continental climate. The primary aim of this hospital-based study was to investigate the XFS frequency and its relationship with systemic diseases such as DM, HT, CAD, smoking, and alcohol intake.

## 2. Materials and Methods

This prospective, single blind, and hospital-based study was conducted at the Sivas Province Numune Hospital Ophthalmology Clinic between May 2013 and October 2013. This study was approved by the Cumhuriyet University Medical Faculty Clinical Studies Ethics Committee and carried out in accordance with the principles of the Helsinki Declaration. A consent form was obtained from all participants.

Cases over the age of 45 years who presented to the clinic were included in the study. Pseudophakic or aphakic cases and those with a history of ocular trauma were excluded. A detailed ophthalmic and systemic history of the patients was obtained and a complete ophthalmic examination was performed. This examination consisted of a visual acuity test with a Snellen chart, slit lamp examination, intraocular pressure (IOP) measurement with the Goldmann applanation tonometer, gonioscopy, visual field analysis, and dilated fundus examination. XFS was diagnosed with the presence of exfoliative material on the lens anterior capsule or iris on slit lamp examination.

Glaucoma was diagnosed with an IOP over 21 mmHg, glaucomatous optic nerve damage, visual field defect, previous history of glaucoma surgery, or the presence of a bleb. Nuclear, cortical, and posterior subcapsular cataracts were rated with the conventional 0–4 grading system. The presence of phacodonesis was evaluated with slit lamp examination. Age-related macular degeneration (AMD) was diagnosed with the findings of this disease on dilated fundus examination. Information on HT, DM, CAD, smoking, and alcohol intake was obtained from the patients with standard queries. The patients were divided into two groups as an XFS group and non-XFS group according to the presence of exfoliative material. The groups were compared in terms of glaucoma, cataract, AMD, phacodonesis, HT, DM, CAD, and frequency of smoking and alcohol intake.

*Statistical Method.* The data were evaluated by means of the SPSS 20.0 program and the descriptive statistics were evaluated using chi-square, *t*-test, and logistic regression analyses. The results are presented in Tables 1–5. A *P* value smaller than 0.05 was considered to be statistically significant. The appropriateness of the model used in logistic regression analysis was evaluated with the Hosmer-Lemeshow goodness-of-fit test and the *χ* ^2^ was 0.95 with *P* > 0.05.

## 3. Results

A total of 2103 cases were evaluated within the scope of the study. All cases were indigenous Central Anatolians.  Table 1 shows the relationship between XFS and age, gender, and the other related factors. A statistically significant relationship was found between XFS and advancing age in both males and females (*P* = 0.001, Table 2).

Unilateral or bilateral XFS was found in 212 cases and the frequency was 10.1%. XFS was found in 140 male patients (14.0%) and in 72 female patients (6.5%) (Table 3). The XFS was bilateral in 108 of the 140 male cases (77.1%) and 53 of the 72 female cases (73.6). XFS was bilateral in 161 (75.9%) and unilateral in 51 (24.1%) of the total number of 212 cases.

Glaucoma was found at a significantly higher frequency in the XFS group than in the non-XFS group. The mean age of the glaucoma cases was 72.0 ± 9.0 years in the XFS group and 62.7 ± 9.9 years in the non-XFS group. The mean IOP was 15.02 ± 5.07 mmHg in the XFS group and 14.17 ± 2.80 mmHg in the non-XFS group. This difference between two groups for IOP was found to be statistically significant (*P* = 0.001, Table 1). The mean IOP in the affected eye (16.85 ± 6.16 mmHg) was found to be significantly higher (14.65 ± 3.82 mmHg) than the other eye in unilateral XFS cases (*P* = 0.037).

The relationship between XFS and cataract, phacodonesis, and AMD was found to be statistically significant (*P* = 0.001). The results are shown in Table 1. According to the results of the logistic regression analysis adjusted for age, a strong relationship was found between XFS and glaucoma, cataract, phacodonesis, and AMD (Table 4).

Logistic regression analysis results as correlated with XFS are presented in Table 5. After adjusting for age, the relationship between XFS and age, male gender, and CAD was found to be statistically significant (*P* = 0.001, *P* = 0.001, and *P* = 0.016, resp.).

## 4. Discussion

XFS frequency varies in different ethnic populations and geographic locations. It is reported to show a wide variation with a frequency of 0% in Eskimos, 0.4% in China, 3.4% in Japan, 9.1% in Jordan, 16.1% in Greece, and 23% in Sweden [14–19]. The XFS frequency in the other two studies conducted in Turkey was 7.2% in the Eastern Mediterranean region in the Yalaz et al. [10] study and 12.2% in Middle Black Sea region in the Cumurcu et al. [11] study. The XFS frequency in our study was found to be 10.1%.

Stein et al. [20] reported an increased XFS frequency with increase in the annual number of sunny days, decreased mean high July temperature, decreased mean January low temperature, and lower elevation above sea level. According to data from the Turkey General Directorate of Meteorology, the mean temperatures of July and January in Sivas Province were 20.2°C and −3.3°C, respectively, and the duration of sunshine was 2421 hours/year with an altitude of 1285 meters. However, a high XFS rate has been reported in Navajo Indians living in Arizona at an altitude of about 1500 m [21]. Sivas has relatively low July and January mean temperatures and also a relatively high altitude.

This study has shown a significant increase in XFS frequency with advancing age along with other studies [16–18]. XFS was also found to be more frequent in male patients than in females and this difference was statistically significant. Yalaz et al. [10] found XFS to be more common in males, but Cumurcu et al. [11] found no relationship between gender and XFS. There are studies reporting a higher XFS frequency in males and in females [18, 19]. However, many studies have reported no significant relationship between gender and XFS [16, 17, 22].

As in previously reported studies, our study also revealed a strong relationship between XFS and glaucoma. Yalaz et al. [10] reported the frequency of glaucoma as 32.1% and Cumurcu et al. [11] as 6.9% in XFS patients. The high frequency of glaucoma in this hospital-based study may be due to the regular follow-ups attended by glaucoma patients. Similarly a high frequency of glaucoma has been found in hospital-based studies with reported rates of 33.1% by Al-Bdour et al. [17], 30.3% by Shazly et al. [23], and 40% by Rao et al. [24]. Kaljurand and Teesalu [25] reported a high frequency of 35.7% for glaucoma in their population-based study although the glaucoma frequency is generally lower in population-based studies [4, 26, 27].

There is a well-known relationship between cataracts and XFS [17, 25, 28]. Regarding the cataract frequency in XFS cases in studies conducted in Turkey, Yalaz et al. [10] found a frequency of 84.6% similar to our result while Cumurcu et al. [11] reported a lower rate at 43.6%. Phacodonesis creates high risk during cataract surgery [29, 30]. The results of this study showed a significant relationship between XFS and phacodonesis. Similarly, Al-Bdour et al. [17] reported a phacodonesis frequency of 7.9% in XFS patients. Cumurcu et al. [11] reported this rate as 14.9%. A statistically significant relationship was found between AMD and XFS in our study, similar to other studies [31, 32].

The Blue Mountains Eye Study reported a significant relationship between XFS and a history of HT, a history of angina or combined angina or myocardial infarction, and a history of stroke [4]. While no relationship was found between XFS and HT or DM in our study, a significant relationship was found with CAD. Of the studies from Turkey, Citirik et al. [12] showed a significant relationship between CAD and XFS, but Emiroglu et al. [13] found no relationship. Many studies have shown a positive relationship between cardiovascular diseases and XFS [4, 33–35]. Furthermore, elevated blood homocysteine levels have been reported to be a risk factor in terms of cardiovascular disease in XFS patients [6]. However, other studies have found no relationship between XFS and cardiovascular disease [36–38]. In conclusion, the relationship between XFS and cardiovascular diseases still remains controversial.

As far as we are aware, this hospital-based study is the first from Turkey to investigate the relationship between XFS and HT, DM, and CAD. The frequency of unilateral and bilateral XFS was 10.1% in this hospital-based study. A statistically significant relationship was found between XFS and advancing age, gender, and CAD.

## Figures and Tables

**Table 1:** XFS-related factors.

	XFS (*n* = 212)	Non-XFS (*n* = 1891)	*χ* ^2^	*P *
Age mean ± SD	71,8 ± 9,2	58,7 ± 9,6		0,001
Mean IOP ± SD	15,02 ± 5,07	14,17 ± 2,80		0,001
Sex (female/male)	72/140	1031/860	32,3	0,001
Glaucoma, *n* (%)	77 (36,3)	65 (3,4)	329,9	0,001
Cataract, *n* (%)	159 (75)	437 (23,1)	252,9	0,001
AMD, *n* (%)	67 (31,6)	121 (6,4)	148,8	0,001
Phacodonesis, *n* (%)	13 (6,1)	7 (0,4)	67,2	0,001
Hypertension, *n* (%)	98 (46,2)	734 (38,8)	4,4	0,036
Diabetes mellitus, *n* (%)	42 (19,8)	404 (21,4)	0,2	0,602
CAD, *n* (%)	46 (21,7)	222 (11,7)	17,0	0,001
Smoking, *n* (%)	16 (7,5)	235 (12,4)	4,4	0,109
Alcohol intake, *n * (%)	1 (0,5)	25 (1,3)	1,1	0,288

**Table 2:** XFS rate according to age and gender.

	Male (XFS+)	Female (XFS+)	Total (XFS+)
45–54	1,6% (5/320)	0,5% (2/433)	0,9% (7/753)
55–64	7,7% (24/312)	3,1% (11/350)	5,3% (35/662)
65–74	22,4% (52/232)	13,0% (30/230)	17,7% (82/462)
75+	43,4% (59/136)	32,0% (29/90)	38,9% (88/226)
Total	**14,0% (140/1000)**	**6,5% (72/1103)**	**10,1% (212/2103)**
	*χ* ^2^ = 162,6 sd = 3 *P* = 0,001	*χ* ^2^ = 146,1 sd = 3 *P* = 0,001	

**Table 3:** XFS rate by sex.

	XFS		Total
	Present	Absent	
Female, *n* (%)	72 (6,5)	1031 (93,5)	1103 (100)
Male, *n* (%)	140 (14)	860 (86)	1000 (100)
Total, *n* (%)	**212 (10,1)**	**1891 (89,9)**	**2103 (100)**

**Table 4:** Relationship between XFS and glaucoma, cataract, phacodonesis, and AMD.

	Glaucoma	Cataract	Phacodonesis	AMD
Non-XFS (reference)	1	1	1	1
XFS odds ratio	10,9	3,7	6,8	2,1
95% CI	7,1–16,8	2,5–5,5	2,4–19,3	1,5–3,1
*P *	0,001	0,001	0,001	0,001

**Table 5:** Associations for XFS using logistic regression analysis.

Variables		OR	95% CI	*P *
Gender	Female (reference)	1		
	Male	1,88	1,34–2,64	0,001

Age	45–54 (reference)	1		
	55–64	5,71	2,50–13,02	0,001
	65–74	21,47	9,69–47,58	0,001
	75+	63,34	28,23–142,08	0,001

Hypertension	Absent (reference)	1		
	Positive	0,89	0,63–1,25	0,493

CAD	Absent (reference)	1		
	Positive	1,66	1,10–2,50	0,016

Diabetes	Absent (reference)	1		
	Positive	0,71	0,47–1,05	0,085

Smoking	Absent (reference)	1		
	Positive	0,87	0,48–1,59	0,659

Alcohol intake	Absent (reference)	1		
	Positive	0,79	0,10–6,49	0,823
